# Supplementary material for: An open-source framework for large-scale, flexible evaluation of biomedical text mining systems
Source: J Biomed Discov Collab. 2008 Jan 29;3:1. doi: 10.1186/1747-5333-3-1 (PMC2276192; doi:10.1186/1747-5333-3-1)
Supplement: Additional file 1 — Evaluation platform source code. The additional file contains the software evaluation framework discussed in this paper. The most current release can be downloaded from SourceForge.net [1]. See the accompanying README file for instructions on installation and use. Also included in the distribution is a collection of UIMA wrappers for some commonly used BioNLP tools and annotated corpora. [file 1747-5333-3-1-S1.zip › bionlp_uima_repository-1.0.1/docs/api/src-html/edu/uchsc/ccp/util/nlp/mention/ClassMentionTypes.html]

```
001    package edu.uchsc.ccp.util.nlp.mention;
002    
003    public interface ClassMentionTypes {
004        
005        /** ClassMention Identifier - if this changes, Annotation DB must be adjusted accordingly */
006        public static final String CLASS_MENTION_TYPE = "class mention";
007        
008        /** for Olga's concept recognizers */
009        public final static String ORGAN = "organ";
010        public final static String DISEASE = "disease";
011        
012        /** Document level Class Mentions */
013        public final static String HTML_PARAGRAPH = "html paragraph";
014        public final static String HTML_PARAGRAPH_START = "paragraph start";
015        public final static String HTML_PARAGRAPH_END = "paragraph end";
016        public final static String HTML_PARAGRAPH_TAG = "paragraph tag";
017        public final static String DOCUMENT_TITLE = "title";
018        public final static String DOCUMENT_SECTION_HEADING = "section heading";
019        public final static String DOCUMENT_SUBSECTION_HEADING = "subsection heading";
020        public final static String DOCUMENT_TABLE_LEGEND = "table caption";
021        public final static String DOCUMENT_FIGURE_LEGEND = "figure caption";
022        
023        
024        /** Syntactic Class Mentions */
025        public static final String TOKEN = "token";
026        public static final String PHRASE = "phrase";
027        public static final String CLAUSE = "clause";
028        public static final String SENTENCE = "sentence";
029    //    public static final String DOCUMENTSECTION = "documentSection";
030        
031    //    public static final String TYPED_DEPENDENCY = "typedDependency";
032        
033        /** Semantic predicates */
034        public static final String EXPRESSION = "expression";
035        
036        /** Semantic Class Mentions */
037        public static final String PROTEIN = "protein";
038        public static final String GENE = "gene";
039        public static final String CELL_TYPE = "cell";
040        public static final String PDG_ACTION = "action";
041        public static final String TRANSPORT_GATED = "gated transport";
042        public static final String GENE_OR_GENE_PRODUCT = "gene or gene product";
043        public static final String MACROMOLECULE = "macromolecule";
044        public static final String NUCLEIC_ACID = "nucleic acid";
045        
046        /* created originally for Bio1 corpus */
047        public static final String CELL_LINE = "cell line";
048        public static final String VIRUS = "virus";
049        public static final String TISSUE = "tissue";
050        public static final String SUBCELLULAR_LOCATION = "subcellular location";
051        public static final String ORGANISM = "organism";
052        public static final String DNA = "DNA";
053        public static final String RNA = "RNA";
054        
055        /* created originally for iProLink corpus */
056        public static final String PROTEIN_ACRONYM = "acronym";
057        public static final String PROTEIN_COMPOUND = "compound protein";
058        public static final String PROTEIN_LONG_FORM = "long form protein";
059        
060        /* for BioEI corpus */
061        public static final String GENE_GENERIC = "generic gene";
062        
063        /** For Penn Bio Tagger */
064        public static final String MALIGNANCY = "malignancy";
065        public static final String VARIATION_EVENT = "variation-event";
066        public static final String VARIATION_TYPE = "variation-type";
067        
068        /** For GENIA tagger */
069        public static final String GENIA_other_artificial_source = "GENIA other artificial source";
070           
071          public static final String PROTEIN_COMPLEX = "protein complex";
072              
073                 
074          public static final String GENIA_other_organic_compound = "GENIA other organic compound";
075              
076          public static final String GENIA_RNA_substructure = "RNA substructure";
077              
078          public static final String GENIA_protein_substructure = "protein substructure";
079              
080          public static final String GENIA_carbohydrate = "carbohydrate";
081              
082          public static final String GENIA_nucleotide = "nucleotide";
083          
084          public static final String GENIA_peptide = "peptide";
085              
086          public static final String GENIA_multi_cell = "multi cell";
087              
088          public static final String GENIA_lipid = "lipid";
089              
090          public static final String GENIA_atom = "atom";
091              
092          public static final String GENIA_RNA_NA = "GENIA RNA N/A";
093              
094          public static final String GENIA_body_part = "body part";
095              
096          public static final String GENIA_polynucleotide = "polynucleotide";
097              
098          public static final String GENIA_inorganic = "inorganic";
099              
100          public static final String GENIA_protein_domain_or_region = "protein domain or region";
101              
102          public static final String GENIA_DNA_domain_or_region = "DNA domain or region";
103              
104          public static final String GENIA_DNA_substructure = "DNA substructure";
105              
106          public static final String GENIA_protein_family_or_group = "protein family or group";
107              
108          public static final String GENIA_cell_component = "cell component";
109              
110          public static final String GENIA_RNA_domain_or_region = "RNA domain or region";
111              
112          public static final String GENIA_other_name = "GENIA other name";
113              
114          public static final String GENIA_DNA_NA = "GENIA DNA N/A";
115              
116          public static final String GENIA_protein_NA = "GENIA protein N/A";
117              
118          public static final String GENIA_protein_subunit = "protein subunit";
119              
120          public static final String GENIA_RNA_family_or_group = "RNA family or group";
121          public static final String GENIA_DNA_family_or_group = "DNA family or group";
122              
123          public static final String GENIA_mono_cell = "mono cell";
124              
125        
126        /** for Mutation project */
127        public static final String MUTATION_EVENT = "mutation event";
128        public static final String SUBSTITUTION = "substitution";
129        public static final String DELETION = "deletion";
130        public static final String INSERTION = "insertion";
131        public static final String BIOLOGICAL_SEQUENCE = "biological sequence";
132        public static final String POLYPEPTIDE_SEQUENCE = "polypeptide sequence";
133        public static final String BIOLOGICAL_SEQUENCE_POSITION = "biological sequence position";
134        public static final String BIOLOGICAL_SEQUENCE_ELEMENT = "biological sequence element";
135        public static final String AMINO_ACID = "amino acid";
136        public static final String ALANINE = "Alanine, Ala, A";
137        public static final String GLYCINE = "Glycine, Gly, G";
138        public static final String VALINE = "Valine, Val, V";
139        public static final String LEUCINE = "Leucine, Leu, L";
140        public static final String ISOLEUCINE = "Isoleucine, Ile, I";
141        public static final String METHIONINE = "Methionine, Met, M";
142        public static final String PHENYLALANINE = "Phenylalanine, Phe, F";
143        public static final String TYROSINE = "Tyrosine, Tyr, Y";
144        public static final String TRYPTOPHAN = "Tryptophan, Trp, W";
145        public static final String SERINE = "Serine, Ser, S";
146        public static final String PROLINE = "Proline, Pro, P";
147        public static final String THREONINE = "Threonine, Thr, T";
148        public static final String CYSTEINE = "Cysteine, Cys, C";
149        public static final String ASPARAGINE = "Asparagine, Asn, N";
150        public static final String GLUTAMINE = "Glutamine, Gln, Q";
151        public static final String LYSINE = "Lysine, Lys, K";
152        public static final String HISTIDINE = "Histidine, His, H";
153        public static final String ARGININE = "Arginine, Arg, R";
154        public static final String ASPARTATE = "Aspartate, Asp, D";
155        public static final String GLUTAMATE = "Glutamate, Glu, E";
156        public static final String BIOLOGICAL_SUBSEQUENCE = "biological subsequence";
157        public static final String POLYPEPTIDE_SUBSEQUENCE = "polypeptide subsequence";
158        
159        /** For Nominalization project */
160        public static final String NOMINALIZATION = "nominalization";
161        public static final String NOMINALIZATION_PHOSPHORYLATION = "Phosphorylation";
162        public static final String NOMINALIZATION_MUTATION = "Mutation";
163        public static final String NOMINALIZATION_FUNCTION = "Function";
164        public static final String NOMINALIZATION_RESPONSE = "Response";
165        public static final String NOMINALIZATION_REGULATION = "Regulation";
166        public static final String NOMINALIZATION_DEVELOPMENT = "Development";
167        public static final String NOMINALIZATION_GROWTH = "Growth";
168        public static final String NOMINALIZATION_TRANSCRIPTION = "Transcription";
169        public static final String NOMINALIZATION_ACTIVATION = "Activation";
170        public static final String NOMINALIZATION_INTERACTION = "Interaction";
171        public static final String NOMINALIZATION_EXPRESSION = "Expression";
172        public static final String NOMINALIZATION_INHIBITION = "Inhibition";
173    
174        /** For MedLEE */
175        public static final String UNDEFINED = "undefined";
176        public static final String DOCUMENT_SECTION = "section";
177        public static final String DOCUMENT_SUBSECTION = "subsection";
178        
179        /** For Species Classification */
180        public static final String TAXON = "taxon";
181        
182    }
```
